# Transcription factor motif activity as a biomarker of muscle aging

**Published:** 2021

**Authors:** Anastasiya Börsch, Mihaela Zavolan

**Affiliations:** Biozentrum, University of Basel and Swiss Institute of Bioinformatics, CH-4056 Basel, Switzerland

**Keywords:** Muscle aging, Gene expression, Motif activity, Regression, Muscle homeostasis, Inflammation

## Abstract

In prior work, we analyzed gene expression profiles of mouse, rat and human gastrocnemius muscles to identify conserved regulators of muscle aging processes. By further comparing data obtained from different muscles we found stronger conservation of aging-related factors at the level of molecular pathways than at the level of individual genes. Here we compared the predictive power of models based on gene expression levels and those based on transcription factor motif activities for an individual’s age. Although somewhat less accurate than models based on gene expression, models based on motif activities achieve good prediction of muscle age, further providing insights into aging-related molecular pathways.

## Abbreviations

ESRRB: Estrogen Related Receptor Beta
ESRRG: Estrogen Related Receptor Gamma
IRF1: Interferon Regulatory Factor 1
IRF2: Interferon Regulatory Factor 2
IRF8: Interferon Regulatory Factor 8
MEF2A: Myocyte-specific Enhancer Factors 2A
MEF2C: Myocyte-specific Enhancer Factors 2C
MEF2D: Myocyte-specific Enhancer Factors 2D
miRNA: microRNA
mRNA: messenger RNA
PC: Principal Component
PI3K: Phosphoinositide 3-kinase
PLSR: Partial Least Squares Regression
RNA: Ribonucleic Acid
RNA-Seq: RNA Sequencing
STAT2: Signal Transducer and Activator of Transcription 2
TBP: TATA-box Binding Protein
TF: Transcription Factor
VIP: Variable Importance in the Projection
YY1: Yin Yang 1
YY2: Yin Yang 2

## Introduction

Model organisms such as rodents are instrumental in uncovering the molecular mechanisms of aging. In recent studies [1,2] we have analyzed the muscle gene expression of aging humans, mice and rats to identify conserved pathways and underlying transcriptional regulators. Two important conclusions of our studies were that there are substantial differences in muscle functionality among individuals of similar chronological age and that the variation in functional and gene expression parameters can be interpreted in terms of a relatively small number of transcriptional regulators. Here we asked whether these can form the basis of a transcriptional clock.

## Materials & Methods

### RNA-Seq data set

We used 181 RNA-Seq samples obtained from human gastrocnemius muscles in the GTEx project (dbGaP accession number phs000424.v8.p2) [3]. Samples from male individuals aged between 22 and 70 years were selected to be: (i) ‘Eligible For Study’; (ii) only from postmortem donors; (iii) only from individuals with the death classification ‘1’ and ‘2’ on the Hardy scale.

### RNA-Seq data processing

Cutadapt v1.9.1 [4] was used to trim the 3’ adapter and poly(A)/poly(T) from the RNA-Seq reads. As the reference transcriptome, we considered protein-coding transcripts with support level 1-3 based on GRCh38 (release 96) and the Ensembl annotation [5]. The assignment of reads to the transcriptome was done with the kallisto software v0.43.1 [6]. All steps were performed with a Snakemake framework [7].

The gene expression level was calculated as the sum of normalized expression levels of transcripts associated with the gene. A gene was considered as expressed if its expression level was at least 1 transcript per million in at least 5 samples. Only expressed genes (~17’500) were considered for the analysis.

### Estimating transcription factor activities

We used the ISMARA tool [8] to estimate the activity of transcription factors (TFs) and miRNAs (~600 motifs) as a function of age in the skeletal muscle. In the analysis we focused on TFs.

### Predicting chronological age from muscle gene expression

We applied the package ‘pls’ in R [9] to construct a linear model based on partial least squares regression [10] taking either gene expression or motif activities in human muscle samples as input to predict chronological age. To rank predictors, ‘variable importance in the projection’ (VIP) scores were calculated [11].

## Results

### Predicting age based on gene expression

**Figure 1:**
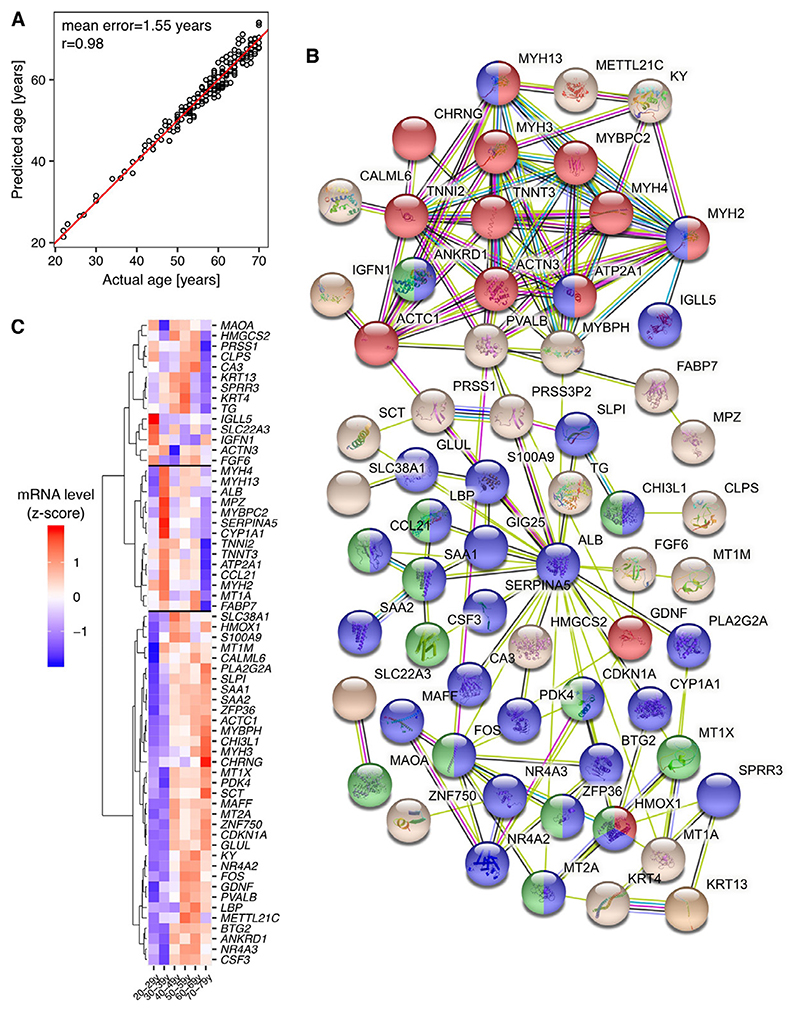
Predicting the age of individuals from muscle gene expression. **A)** Scatter plot depicting the actual vs. predicted age, each dot corresponding to one sample. Red - the reference line with slope 1 and intercept 0. ‘r’ - Pearson correlation coefficient. **B)** Top 100 predictor genes visualized in STRINGdb [18]. Only nodes already known to be involved in protein-protein interactions are shown. Nodes that significantly enriched (FDR<0.05) specific biological processes are marked in red - ‘muscle system process’, blue - ‘response to stress’, and green - ‘cellular response to cytokine stimulus’. **C)** Heatmap depicting z-scores of the expression level of top predictor genes (from panel B) in samples from individual age groups. The mean value within age groups was used.

To set a baseline for the prediction power of gene expression-based models, we used partial least squares regression (PLSR) to construct a linear model taking the muscle gene expression as input to predict chronological age [10]. To train the model, we used the nine principal components (PCs) that together explained more than 50% of the variance in gene expression levels across all samples. The resulting model predicted the age of individuals with a mean error of 1.55 years and Pearson correlation coefficient r=0.98 between the actual and predicted ages (Figure 1A). Cross-validation by randomly splitting samples 80%-20% for model training and testing 100 times gave a mean absolute prediction error of 1.47 years for the training data sets, and 6.95 years for the 4-fold smaller validation data sets (Figure S1A).

For each cross-validation run we also collected top 100 predictor genes based on the ‘variable importance in the projection’ (VIP) scores calculated during model training [11], and then computed the mean VIP score for each gene that appeared at least once among top predictors. Submitting the 100 genes with the highest mean score to STRINGdb [12] for functional analysis we found that the encoded proteins form two major hubs (Figure 1B), one corresponding to the Gene Ontology annotation ‘muscle system process’ (red), and the other to ‘response to stress’ (blue) and ‘cellular response to cytokine stimulus’ (green). Their mRNA levels either increased or decreased abruptly at advanced age (Figure 1C). Thus, gene expression levels in the muscle are highly indicative of the individual’s age.

### Predicting individual age based on motif activities

The functional relationships between age-predicting genes indicate the action of transcription factors (TFs) that coordinate specific biological processes during aging. We thus asked whether TF activity may also serve as a reliable predictor of age.

**Figure 2:**
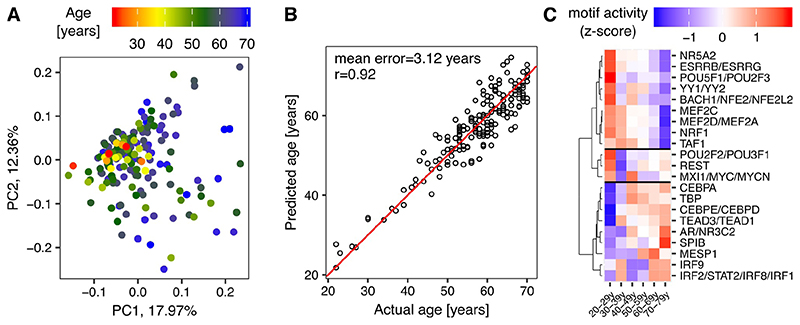
Predicting individual age from TF motif activities. **A)** Principal component analysis of motif activities. Each dot corresponds to one sample, colors indicate the age of individuals from which the samples were obtained. The numbers associated with the PCs indicate the fraction of the variance in motif activities across samples that is captured by the corresponding PC. **B)** Scatter plot depicting the actual age of individuals vs. the age predicted by the model based on motif activities in the muscle, each dot corresponding to one sample. Red - the reference line with slope 1 and intercept 0. ‘r’ - Pearson correlation coefficient. **C)** Heatmap depicting z-scores of top predictor motif activities. The mean motif activity within age groups was used.

To infer the activity of motifs corresponding to TFs during muscle aging, we applied the ISMARA tool [8] to the RNA-Seq data set. Interestingly, the principal component analysis of motif activities reveals the same progressive transition from adult to sarcopenic phases (Fig. 2A here vs. Fig. 2C in [2]).

Further, we followed the steps described in the previous section to model the age based on motif activities. To set up the model, we used eight PCs that together explained more than 50% of the variance in motif activities in all samples. Although the number of features was significantly smaller than when using gene expression (~600 motifs vs. ~17’500 genes), the model predicted the age of individuals quite well, with the mean error 3.12 years and Pearson’s correlation coefficient r=0.92 (Figure 2B). As before, we estimated the performance of the model by cross-validation. Prediction errors for training and testing data sets as well as top 10 predictors defined by their VIP scores were collected for each validation round. The mean absolute prediction error was 2.67 years in the training data sets, and 8 years for the 4-fold smaller validation data sets (Figure S1B). The union of top predictors from the cross-validation procedure are shown in Figure 2C. These motifs represent potential muscle biomarkers of human aging, some of them having already been associated with muscle functionality.

Identified in all cross-validation rounds were the TATA-box binding protein (TBP), the myocyte-specific enhancer factors MEF2D/MEF2A and MEF2C and the interferon regulatory factors IRF2/STAT2/IRF8/IRF1. While TBP is necessary for muscle differentiation [13], it also regulates numerous inflammation-related targets (Figure S2A). Increased activity of TBP and IRF2/STAT2/IRF8/IRF1, factors that mediate immune and inflammatory responses in literally all mammalian tissues [14], likely reflects the aging-related inflammatory syndrome (Figure S2B). In contrast, the activity of MEF factors, which regulate expression of structural constituents of the muscle tissue such as sarcomere units Z-discs and M-bands (Fig. S2C, D), decreases in aging.

The analysis also uncovered that the activity of motifs associated with C/EBP family TFs CEPBE, CEBPD and CEBPA increases during muscle aging (Figure 2C). TFs of this family are known to interact with the activating transcription factor 4 (ATF4) and regulate skeletal muscle atrophy [15].

Taken together, muscle motif activities serve as reliable predictors of age in humans and provide insights into the molecular pathways that are involved in aging processes.

## Discussion

Regression models and deep-learning approaches have been previously applied to identify predictors of muscle age from gene expression [16]. Here we applied PLSR to predict muscle age from either gene expression or the activity of transcription regulatory motifs in the gastrocnemius muscle. We selected PLSR, because it has been designed for situations when there are many, possibly correlated, predictor variables and relatively few samples [10], as is the case here, with ~17’500 genes and ~180 samples. The resulting model accurately predicted the age of muscle samples with the mean error of 1.55 years and Pearson correlation coefficient r=0.98 between the actual and predicted ages (Figure 1A). The majority of top predictors are known to be involved in protein-protein interacting with each other and in processes regulating muscle homeostasis and inflammation (Figures 1B and 1C). Since these processes belong to the main pathophysiological pathways contributing to physical frailty and sarcopenia [17], genes that enriched these processes may be of interest for future studies as biomarkers of muscle aging.

Our previous studies [1,2] indicated that muscle aging may not involve precisely the same genes in all muscles, but rather similar pathways. Thus, to identify upstream regulators of these pathways, we inferred the activity of transcription factor (TF) motifs in muscle samples using ISMARA [8]. Modeling individual’s age from motif activities also yielded high-accuracy predictors with mean error of 3.15 years and Pearson correlation coefficient r=0.92 between the actual and predicted ages (Figure 2B). The difference in accuracy between the two models may be due to target predictions being available only for a subset of TFs, whereas the gene expression was estimated based on the entire transcriptome.

The predictors of both models can be directly related, as top predictive motif activities correspond to known regulators of muscle homeostasis (e.g. MEF2D/MEF2A and MEF2C) and inflammation (e.g. IRF2/STAT2/IRF8/IRF1). The role of predictive TFs whose activity decreases during muscle aging not only in humans, but also mice and rats, namely ESRRB/ESRRG, YY1/YY2 and NR5A2 was discussed before [2].

## Conclusion

Taken together, our results demonstrate that aging affects conserved pathways, rather than effector genes. Motif activities can be used to model the age of muscle tissue and top predictors can be further studied as potential targets to improve muscle health during aging.

## Supplementary Material

Supplementary Material
