# Supplementary material for: Transcription factor motif activity as a biomarker of muscle aging
Source: Am J Aging Sci Res. Author manuscript; Available in PMC 2022 Jan 25. (PMC7612261)
Supplement: Supplementary Material [file EMS140731-supplement-Supplementary_Material.docx]

Supplementary Material
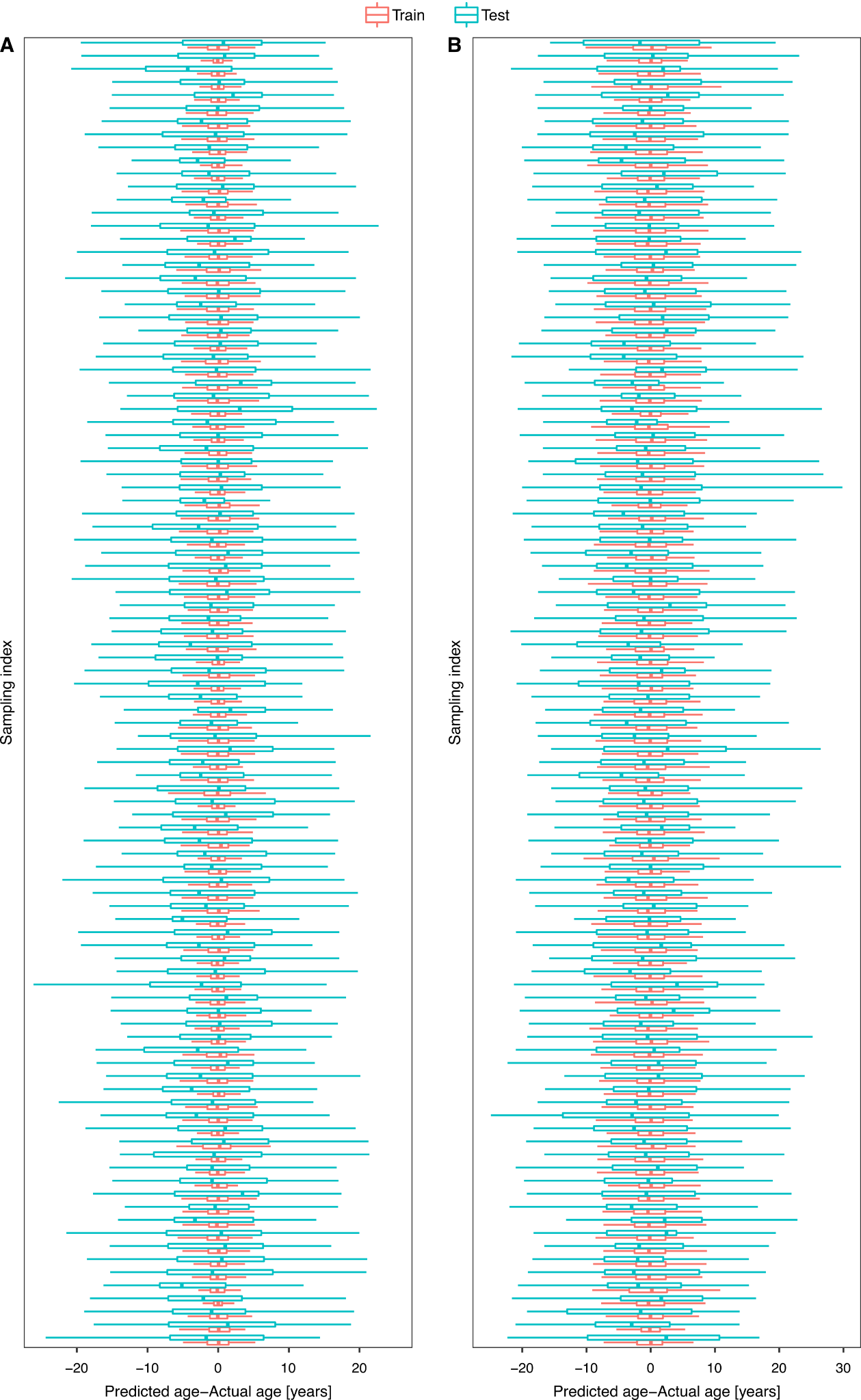


**Figure S1.** Results of cross-validation of the linear model predicting the age of human muscle samples. A) Based on the gene expression in these samples. B) Based on TF motif activities in these samples. Boxplots summarize prediction errors obtained for training and testing data sets for 100 cross-validation rounds.


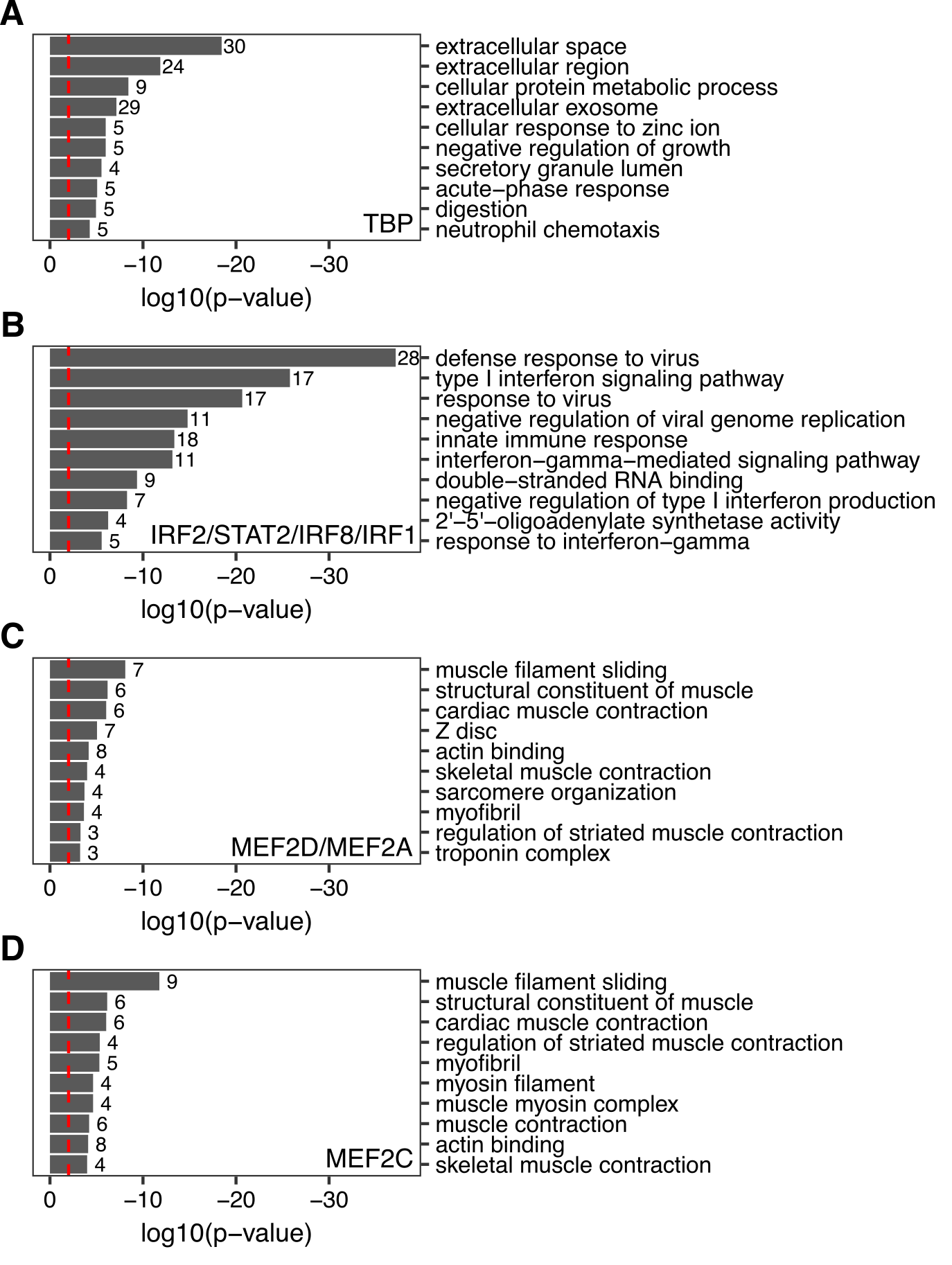


**Figure S2.** Gene ontology (GO) analysis of top 100 target genes of top motifs. A) TBP (623 target genes in total). B) IRF2/STAT2/IRF8/IRF1 (1021 target genes in total). C) MEF2D/MEF2A (434 target genes in total). D) MEF2C (285 target genes in total). Top 10 most enriched GO terms for the top 100 target genes of the TFs are shown. GO analysis was performed in DAVID (<https://david.ncifcrf.gov/>). Red dashed lines indicate the significance threshold (p-value<0.01). The numbers next to the bars denote how many genes were attributed to an enriched GO term.
